# An Improved ELOS Guidance Law for Path Following of Underactuated Unmanned Surface Vehicles

**DOI:** 10.3390/s24165384

**Published:** 2024-08-20

**Authors:** Shipeng Wu, Hui Ye, Wei Liu, Xiaofei Yang, Ziqing Liu, Hao Zhang

**Affiliations:** College of Automation, Jiangsu University of Science and Technology, Zhenjiang 212000, China; 221110303122@stu.just.edu.cn (S.W.); yehuicc@just.edu.cn (H.Y.); yxfei_0809@just.edu.cn (X.Y.); 13395424593@163.com (Z.L.); 221110303127@stu.just.edu.cn (H.Z.)

**Keywords:** ELOS, path following, unmanned surface vehicles, UWB

## Abstract

In this paper, targeting the problem that it is difficult to deal with the time-varying sideslip angle of an underactuated unmanned surface vehicle (USV), a line–of–sight (LOS) guidance law based on an improved extended state observer (ESO) is proposed. A reduced-order ESO is introduced into the identification of the sideslip angle caused by the environmental disturbance, which ensures a fast and accurate estimation of the sideslip angle. This enables the USV to follow the reference path with high precision, despite external disturbances from wind, waves, and currents. These unknown disturbances are modeled as drift, which the modified ESO-based LOS guidance law compensates for using the ESO. In the guidance subsystem incorporating the reduced-order state observer, the observer estimation and track errors are proved uniformly ultimately bounded. Simulation and experimental results are presented to validate the effectiveness of the proposed method. The simulation and comparison results demonstrate that the proposed ELOS guidance can help a USV track different types of paths quickly and smoothly. Additionally, the experimental results confirm the feasibility of the method.

## 1. Introduction

Unmanned surface vehicles (USVs) have seen widespread application in various domains, emerging as a focal point in intelligent marine equipment research in recent years. In the dynamic and unpredictable ocean environment, the capability to accurately, swiftly, and effectively track a designated path is a crucial technology for ensuring the safety and successful completion of USV missions.

There has been extensive research on path following, which is firstly focused on wheeled mobile robots (WMR) in [1]. The more common solutions for path following are discussed in detail [2]. The authors propose a strategy that involves projecting the actual position of vehicle onto the reference geometrical path. This method ensures that a virtual vehicle is always positioned at the point on the path closest to the real vehicle. This is performed using the moving path frame, which is established based on the Serret-Frenet (SF) equations. This path frame is the path tangential coordinate system at the exact path projection point. On this basis, the path following problem is solved within the path following error space. However, this approach has significant drawbacks. If the controlled object is always located at the origin of the osculated circle, then the projected point of the controlled object will move infinitely fast on the path [3]. Consequently, the SF kinematics exhibit a singularity at this point. Instead of always attaching the SF frame to the precise point on the path closest to the WMR, the SF frame allows for dynamic evolution according to a properly defined time function. This adjustment removes the singularities [4].

In order to simplify the controller design, the path following the control system of underactuated USV is divided into two main parts: the guidance subsystem (GS) and the control subsystem (CS). Once the reference path and environment data are obtained, the GS can provide reference signals for the CS. The CS then follows these reference signals to ensure precise tracking. Typical guidance methods include a vector field [5,6], line-of-sight guidance [7,8], pure pursuit guidance, constant bearing, etc.

The LOS method is widely employed in USV path following control due to its simplicity and reliable convergence. The heading angle command based on line–of–sight guidance law refers to the rich experience of the navigator. In the GS of many USVs, the proportional line–of–sight guidance law is used for path following, and the proportional gain is inversely proportional to the look–ahead distance. The uniform global asymptotic stability of the proportional LOS guidance law was proven by Petterse et al. [9]. In [10], it has been demonstrated that the origin of the path following the error dynamics system exhibits uniform semi-global exponential stability. This ensures robustness and strong convergence properties against disturbances [11]. 

Despite the effectiveness and widespread use of proportional LOS guidance laws, they are limited when vehicles are subjected to drift forces from winds, waves, and ocean currents, leading to significant tracking errors during path following. A straightforward method to compensate for sideslip angle involves measuring it using accelerometers, GPS, and other sensors. However, this approach is susceptible to sensor noise and measurement inaccuracies. A more effective solution is the integral LOS (ILOS) method [12], which improves the robustness of the system [13]. The closed-loop following system has been proven to possess globally uniformly asymptotic stability and locally uniformly exponential stability [14]. Adaptive sideslip compensation can be achieved in the integral LOS guidance law by replacing the integral state with a parameter [15]. Additionally, in [13] the direct and indirect adaptive control laws are developed for ILOS path following. Nie et al. [16] have introduced an adaptive ILOS guidance law, which computes the desired yaw angle while simultaneously estimating time-varying ocean currents and the sideslip angle.

In addition to integral LOS, predictor-based LOS methods have been proposed to deal with unknown sideslip angle. Liu et al. [17] constructed a state predictor first, and subsequent studies explored various predictor-based LOS guidance laws [18,19]. Particularly, Wang et al. [20] developed a predictor specifically to estimate the sideslip within a fixed time. In [21], to address scenarios with an unknown time-varying large sideslip angle, which cannot be linearized using small-angle approximation, a finite-time sideslip observer was developed to estimate the varying sideslip angle accurately. This approach led to the proposal of hyperbolic–tangent LOS guidance laws in [22], where a virtually desired sideslip angle is defined to precisely capture the tangent nonlinearity identified by the finite-time sideslip observer.

Another design approach is the ELOS guidance law [23], which utilizes a reduced-order ESO to estimate the sideslip angle. Nie et al. [24] propose a finite time ESO to accurately observe the unknown velocity and a finite time LOS guidance law based on these velocity observations for investigation to obtain the desired heading angle. In [25], an ESO with a prescribed time was used to accurately determine the sideslip angle, especially when following a curved path or when in the presence of time-varying wind and wave disturbances. A comparative analysis of the ALOS, ELOS, and ILOS guidance laws for path following can be found in [26,27]. Weng et al. [28] developed a data-driven sideslip observer-based line–of–sight (DLOS) guidance law, and the sideslip is compensated for by using a data-driven sideslip observer when the surge speed is unmeasurable.

In the methods mentioned above, it is often assumed that the sideslip angle remains small. Typically, the literature assumes the sideslip angle to be less than 5°, allowing for approximations sinβ≈β and cosβ=1 to hold. Alternatively, the sideslip angle may be considered constant, implying $\dot{\beta }=0$. However, wind and ocean currents vary over time in natural water environments. This variability causes the time-varying sideslip angle of the USV to exceed 10° due to wind and wave current disturbances. Furthermore, most of these methods have primarily been validated theoretically, lacking practical experimentation.

The effect of the guidance law is limited by the accuracy of the position measurement. In terms of the positioning of the USV, GPS signals are susceptible to interference [29], and weather and light intensity [30] affect the lidar and camera, respectively. Many methods have been proposed to improve the positioning accuracy. Yan et al. [31] proposed a navigation accuracy compensation algorithm for low-cost USVs. Moreover, in [32], a cooperative navigation algorithm with observability and trilateral positioning method was proposed to compensate for the estimated position errors for EKF. Ultra-wideband (UWB) technology has the advantages of stable signal, strong anti-multipath effect, simple structure, easy deployment, and simple principle [33]. It is independent of weather conditions and provides high positioning accuracy. UWB positioning devices are not affected by channel fading, exhibit robust interference resistance, and do not interfere with other equipment. Thus, applying UWB positioning technology for localizing USVs in nearshore areas is highly promising. UWB technology has already been successfully applied across different domains, such as indoor positioning [34], positioning of unmanned aerial vehicles [35], autonomous driving for automobiles [36], and robot localization systems [37].

Based on this, an ESO is proposed to estimate the sideslip angle of the USV, which is then substituted into the LOS guidance law. In addition, this study also explores the application of UWB technology in USV positioning. The following is a summary of the main contributions of this study:

(1) The reduced order ESO is improved to estimate the sideslip angle, and the small angle approximation principle of the sideslip angle is eliminated. The structure of the observer is simplified, which estimates the time-varying sideslip angle quickly and accurately. 

(2) Different from the traditional ELOS guidance law, which compensates for the sideslip angle through the saturated arctangent function, the improved ELOS in this paper uses a different law to calculate the desired heading angle. The improved guidance law has almost the same tracking performance as the traditional ELOS under normal conditions. However, in simulations and experiments, the modified ELOS guidance law shows superior tracking performance for rapidly changing sideslip angles caused by environmental disturbances. In the simulation, compared to ILOS, the improved ELOS reduces average cross–track error by 24.9%, reduces energy consumption by 5.2%, and improves smoothness by 3.4%. Compared with the classical ELOS, the improved ELOS reduces the cross–track error by 19.6%, reduces energy consumption by 7%, and improves the smoothness by 7.7%.

(3) The application of the UWB for positioning USVs is achieved within the context of ship navigation. A scheme to meet centimeter-level accuracy based on UWB systems has been developed. This technology is not affected by environmental weather, and has the advantages of a simple principle, ease of use, and high accuracy.

## 2. Materials and Methods

**Figure 1:**
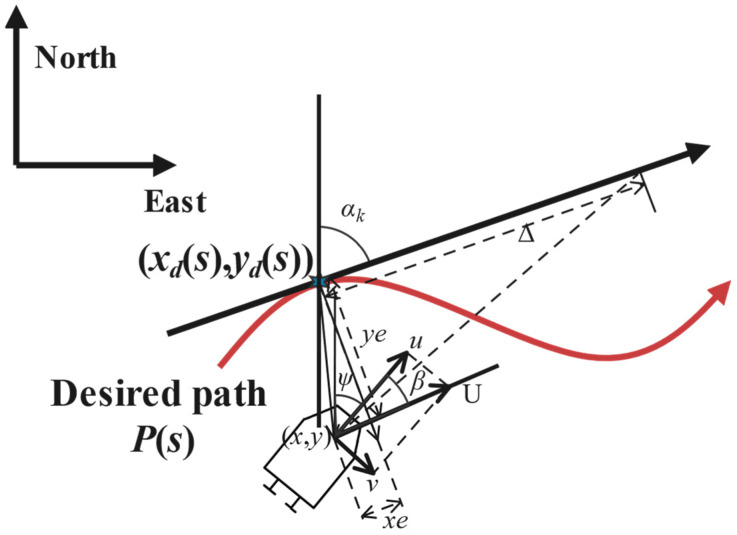
Geometric illustration of the LOS guidance law.

According to [38], the kinematic equations of the 3-DOF USV can be expressed as

(1) $$\left\{\begin{array}{c} \dot{x}=u\cos\psi -\upsilon \sin\psi \\ \dot{y}=u\sin\psi +\upsilon \cos\psi \\ \dot{\psi }=r \end{array}\right.$$

where x and y denote the cross and vertical coordinates of the vehicle, and ψ represents the heading angle in the inertial coordinate system, respectively; u, v, and r represent the surge velocity, sway velocity, and yaw rate of the USV in the body-fixed (BF) coordinate system, respectively (see Figure 1).

Figure 1 illustrates the geometric depiction of the LOS-guided path following the USV. By introducing a variable *s*, a path dependent on *s* can be defined in Figure 1. Therefore, any point along this path can be denoted as $\left(x_{k}\left(s\right),y_{k}\left(s\right)\right)$, where *s* serves as the path variable. Subsequently, the path–tangential angle $\alpha_{k}\left(s\right)$ at this point can be calculated via $\alpha_{k}\left(s\right)=atan2\left(y_{k}^{\prime }\left(s\right),x_{k}^{\prime }\left(s\right)\right)$ with $x_{k}^{\prime }\left(s\right)=\partial x_{k}/\partial s$ and $y_{k}^{\prime }\left(s\right)=\partial y_{k}/\partial s$. In this context, the path–tangential (PT) reference frame can be obtained by clockwise rotation of the inertial coordinate system by the angle $\alpha_{k}\left(s\right)$.

Next, $x_{e}$ and $y_{e}$ are the path following error in the PT reference frame, denoted by:

(2) $$\left[\begin{array}{c} x_{e}\\ y_{e} \end{array}\right]={\left[\begin{array}{c} {\cos\alpha }_{k}-{\sin\alpha }_{k}\\ {\sin\alpha }_{k}\;{\cos\alpha }_{k} \end{array}\right]}^{T}\left[\begin{array}{c} x-x_{k}\left(s\right)\\ y-y_{k}\left(s\right) \end{array}\right]$$

Taking the time derivative of $x_{e}$ and $y_{e}$, the dynamics of $x_{e}$ and $y_{e}$ are obtained using:

(3) $$\left\{\begin{array}{l} {\dot{x}}_{e}=U\cos(\psi -\alpha_{k}+\beta )+{\dot{\alpha }}_{k}y_{e}-u_{p}\\ \begin{array}{c} {\dot{y}}_{e}=U\sin(\psi -\alpha_{k}+\beta )-{\dot{\alpha }}_{k}x_{e}\\ \dot{s}=\frac{u_{p}}{\sqrt{{{x^{\prime }}_{k}}^{2}+{{y^{\prime }}_{k}}^{2}}} \end{array} \end{array}\right.$$

where $U=\sqrt{u^{2}+v^{2}}$ denotes the practical velocity of the USV, and β=atan2(v,u) represents the sideslip angle due to the influence of the sway velocity v. It should be noted that the sideslip angle β represents the direction of the velocity vector U with respect to the BF frame, which is usually unknown in practical situations. Since the underactuated USV cannot directly control the force in the roll direction, it is likely to produce large tracking errors under the influence of the sideslip angle. Therefore, in order to ensure that the underactuated USV can accurately track the path, it is essential to compensate for the sideslip angle.

This paper aims to design a guidance law for the kinematical equation of the USV described by (1), such that the USV converges to and follows a specified geometric path $\left(x_{k}\left(s\right),y_{k}\left(s\right)\right)$ from any initial position, even in the presence of unknown varying sideslip angles. The design goal of the path following is formulated as:

(4) $$\underset{t\to \infty }{\lim}x_{e}\leq \delta ,\underset{t\to \infty }{\lim}y_{e}\leq \delta$$

for some small constants δ.

**Remark** **1.** *Unlike existing achievements that focus on the constant sideslip angle [13,23], the sideslip angle in this paper is time-varying during the USV path following. The approach offers a more practical guidance strategy for the USV path following the problem.*

## 3. The Design and Analysis of the Improved ELOS Guidance Law

### 3.1. Sideslip Angle Estimation

The sideslip angle is often unknown due to the lack of direct measurement. Although many methods have been proposed to compensate for the constant sideslip angle, the sideslip angle of an underactuated USV can also be affected by time-varying wind and wave current disturbances. Furthermore, the sideslip angle of the USV when moving along a curved path changes even when the external disturbance is assumed to be constant. Hence, a reduced-order ESO is used to estimate the sideslip angle because

(5) $$\left\{\begin{array}{c} U\cos\beta =u\\ U\cos\beta =v \end{array}\right.$$

Rewrite the first and second formulas in (3) as:

(6) $$\left\{\begin{array}{c} {\dot{x}}_{e}=u\cos\left(\psi -\alpha_{k}\right)-u\sin\left(\psi -\alpha_{k}\right)\tan\beta +{\dot{\alpha }}_{k}y_{e}-u_{p}\\ {\dot{y}}_{e}=u\sin\left(\psi -\alpha_{k}\right)+u\cos\left(\psi -\alpha_{k}\right)\tan\beta -{\dot{\alpha }}_{k}x_{e} \end{array}\right.$$

Let

(7) $$g=u\cos\left(\psi -\alpha_{k}\right)tan\beta$$

Hence

(8) $$\left\{\begin{array}{l} {\dot{x}}_{e}=u\cos\left(\psi -\alpha_{k}\right)-u\sin\left(\psi -\alpha_{k}\right)\tan\beta +{\dot{\alpha }}_{k}y_{e}-u_{p}\\ {\dot{y}}_{e}=u\sin\left(\psi -\alpha_{k}\right)+g-{\dot{\alpha }}_{k}x_{e} \end{array}\right.$$

**Assumption** **1.** *The surge velocity* u *is measured and bounded, i.e.,* $0<u_{min}<u<u_{max}$*. A positive constant* $g_{*}$ *meets the conditions that* $\left|g\right|\leq g_{*}$ *and* $\left|\dot{g}\right|\leq g_{*}$.

Considering that g includes the unknown sideslip angle β, to calculate the estimation of the time varying β, the reduced order ESO [23] is modified as:

(9) $$\left\{\begin{array}{l} \dot{p}=-k_{g}p-k_{g}^{2}y_{e}-k_{g}\left[u\sin\left(\psi -\alpha_{k}\right)-{\dot{\alpha }}_{k}x_{e}\right]\\ \hat{g}=p+k_{g}y_{e} \end{array}\right.$$

where p represents the auxiliary state of the observer, $k_{g}$ represents the gain, and $\hat{g}$ is the estimation of g. Let the initial values of the observer be $\hat{g}\left(t_{0}\right)=0$ by setting $p\left(t_{0}\right)=-k_{g}y_{e}\left(t_{0}\right)$.

**Remark** **2.** *The reduced order ESO is first proposed to estimate the sideslip angle for LOS guidance law in [23]. A similar ESO is designed in [39]. The reduced-order ESO is originally investigated to estimate the lumped disturbances in [40]. Interestingly, according to the disturbance observer design method [41], a similar observer can also be obtained.*

As $\hat{g}$ is known by the ESO, and the calculation of the estimated sideslip angle $\hat{\beta }$ can be determined by

(10) $$\hat{\beta }=arctan\frac{\hat{g}}{u\cos\left(\psi_{d}-\alpha_{k}\right)}$$

Investigating the convergence of the reduced order ESO, the estimation error of the sideslip angle is defined as $\tilde{g}=g-\hat{g}$, whose derivative can be expressed using (8) and (9):

(11) $$\dot{\tilde{g}}=\dot{g}+k_{g}p+k_{g}^{2}y_{e}+k_{g}\left[u\sin\left(\psi_{d}-\alpha_{k}\right)-{\dot{\alpha }}_{k}x_{e}\right]\;-k_{g}\left[u\sin\left(\psi -\alpha_{k}\right)+g-{\dot{\alpha }}_{k}x_{e}\right]\;=\dot{g}-k_{g}\tilde{g}$$

Then, it gives

(12) $$\frac{d}{dt}\left|\tilde{g}\right|=\left|\dot{g}\right|-k_{g}\left|\tilde{g}\right|$$

Therefore, it can be concluded that

(13) $$\left|\tilde{g}\right|\leq e^{-k_{g}t}\left|\tilde{g}\left(0\right)\right|+\frac{g_{*}}{k_{g}}$$

The estimation error $\tilde{g}$ shows an exponential decreasing trend. Lemma 4 in [23] provides results about the relationship between the bound on this error $\tilde{g}$ and the bandwidth of the reduced order ESO. According to the theoretical findings [23], the estimation error $\tilde{g}$ can be minimized to nearly zero by enhancing the bandwidth of the ESO within a short transient process $t>t_{0}+max\left\{0,Ink_{g}/k_{g}\right\}$. Moreover, the method in [23,42] can be used to demonstrate the input–to–state stability of (11).

**Remark** **3.** *Ref. [21] indicates that the increased bandwidth of the reduced-order ESO results in a smaller observation error over* $t\in \left[t_{0},\infty )\right.$*. Moreover, the error converges to a small value after a brief transient period of duration* $max\left\{0,Ink_{g}/k_{g}\right\}$*. Nevertheless, practical limitations often restrict the bandwidth of ESO due to measurement noise. Therefore, achieving satisfactory performance in the presence of measurement noise involves tuning the observer’s bandwidth carefully, balancing between estimation accuracy and sensitivity to noise.*

### 3.2. Guidance Law Design

**Figure 2:**
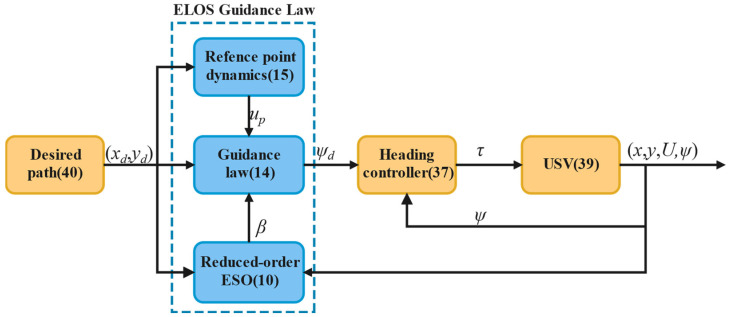
Block diagram of the path following the system with ELOS guidance.

The improved ELOS guidance law comprises a reduced order ESO, a motion law of virtual vehicles, and a guidance law, illustrated in Figure 2. This ELOS guidance subsystem operates independently of the control system and is integrated with a heading autopilot.

In a different form [23], the guidance law is formulated as:

(14) $$\psi_{d}=\alpha_{k}\left(s\right)-\hat{\beta }+\arctan\left(-\frac{y_{e}}{\Delta }\right)$$

where Δ is the look-ahead distance; Δ is about 5 to 8 times the length of the USV.

**Remark** **4.** *The guidance law (14) is different from the classical and adaptive ILOS guidance laws, in that the integral state is replaced by the estimated sideslip angle.*

For the first equation of (8), to stabilize $x_{e}$, the virtual input $u_{p}$ is selected as:

(15) $$u_{p}=u\cos\left(\psi -\alpha_{k}\right)-u\sin(\psi -\alpha_{k})\tan(\hat{\beta })+\kappa x_{e}$$

where κ>0 is the tuning parameter.

From (3) and (15), the updated law for s is derived as:

(16) $$\dot{s}=\frac{u\cos\left(\psi -\alpha_{k}\right)-u\sin(\psi -\alpha_{k})\tan(\hat{\beta })+\kappa x_{e}}{\sqrt{{{x^{\prime }}_{k}}^{2}\left(s\right)+{{y^{\prime }}_{k}}^{2}\left(s\right)}}$$

By substituting Equation (15) into the first equality of (8), we obtain

(17) $${\dot{x}}_{e}=-\kappa x_{e}+{\dot{\alpha }}_{k}y_{e}$$

**Assumption** **2.** *The heading autopilot can accurately track the desired heading angle* $\psi_{d}$*, i.e.,* $\psi_{d}=\psi$*.*

Based on Assumption 2, one can obtained as follows:


(18) $$\psi -\alpha_{k}=\psi_{d}-\alpha_{k}=-\hat{\beta }+\arctan\left(-\frac{y_{e}}{\Delta }\right)$$

since


(19) $$\left\{\begin{array}{l} \sin\left({tan}^{-1}\left(-\frac{y_{e}}{∆}\right)\right)=-\frac{y_{e}}{\sqrt{{∆}^{2}+y_{e}^{2}}}\\ \cos\left({tan}^{-1}\left(-\frac{y_{e}}{∆}\right)\right)=\frac{∆}{\sqrt{{∆}^{2}+y_{e}^{2}}} \end{array}\right.$$

This gives

(20) $$\sin\left(\psi -\alpha_{k}\right)=-c_{1}\sin\hat{\beta }-c_{2}\cos\hat{\beta }$$

(21) $$\cos\left(\psi -\alpha_{k}\right)=c_{1}\cos\hat{\beta }-c_{2}\sin\hat{\beta }$$

where $c_{1}=\frac{∆}{\sqrt{{∆}^{2}+y_{e}^{2}}}$, $c_{2}=\frac{y_{e}}{\sqrt{{∆}^{2}+y_{e}^{2}}}$.

Substituting (21) into (7), it gives

(22) $$\begin{array}{c} \hat{g}=u\cos\left(\psi_{d}-\alpha_{k}\right)tan\hat{\beta }\\ =u\left(c_{1}\cos\hat{\beta }-c_{2}\sin\hat{\beta }\right)tan\hat{\beta }\\ =uc_{1}\sin\hat{\beta }-\frac{uc_{2}\sin^{2}\hat{\beta }}{\cos\hat{\beta }} \end{array}$$

By substituting Equation (20) into the second equality of (8), it gives

(23) $$\begin{array}{c} {\dot{y}}_{e}=u\sin\left(\psi -\alpha_{k}\right)+g-{\dot{\alpha }}_{k}x_{e}\\ =u\left(-c_{1}\sin\hat{\beta }-c_{2}\cos\hat{\beta }\right)+g-{\dot{\alpha }}_{k}x_{e}\\ =-\hat{g}-\frac{uc_{2}\sin^{2}\hat{\beta }}{\cos\hat{\beta }}-uc_{2}\cos\hat{\beta }+g-{\dot{\alpha }}_{k}x_{e}\\ =-\frac{1}{\cos\hat{\beta }}\frac{uy_{e}}{\sqrt{{∆}^{2}+y_{e}^{2}}}+\tilde{g}-{\dot{\alpha }}_{k}x_{e} \end{array}$$

Finally, the error dynamics (8) turn into

(24) $$\left\{\begin{array}{c} {\dot{x}}_{e}=-\kappa x_{e}+{\dot{\alpha }}_{k}y_{e}\\ {\dot{y}}_{e}=-\frac{1}{\cos\hat{\beta }}\frac{uy_{e}}{\sqrt{{∆}^{2}+y_{e}^{2}}}+\tilde{g}-{\dot{\alpha }}_{k}x_{e} \end{array}\right.$$

**Theorem** **1.** *Subsystem (24), considered as a system with* $x_{e}$ *and* $y_{e}$ *as the states, and* $\hat{g}$ *and* β *as the inputs, is uniformly ultimately bounded under conditions* $c_{min}-\frac{1}{2\epsilon_{1}}>0,k_{g}-\frac{\epsilon_{1}}{2}-\frac{\epsilon_{2}}{2}>0$ *and* $\epsilon_{1}>0$*,* $\epsilon_{2}>0$*.*

**Proof** **of** **Theorem** **1.** Consider Lyapunov function candidate

(25) $$V_{2}=\frac{1}{2}x_{e}^{2}+\frac{1}{2}y_{e}^{2}+\frac{1}{2}{\tilde{g}}^{2}$$

The derivative of $V_{2}$ with respect to (25) satisfies

(26) $${\dot{V}}_{2}=-\kappa x_{e}^{2}-cy_{e}^{2}+y_{e}\tilde{g}+\dot{g}\tilde{g}-k_{g}{\tilde{g}}^{2}$$

where $c=\frac{1}{\cos\hat{\beta }}\frac{u}{\sqrt{{∆}^{2}+y_{e}^{2}}}$, since $\cos\hat{\beta }\in \left(0,1]\right.$

(27) $$-c<{-c}_{min}=-\frac{u_{min}}{\sqrt{{∆}^{2}+y_{e}^{2}}}$$

using the inequality

(28) $$y_{e}\tilde{g}\leq \frac{1}{2\epsilon_{1}}y_{e}^{2}+\frac{\epsilon_{1}}{2}{\tilde{g}}^{2}$$

(29) $$\dot{g}\tilde{g}\leq \frac{1}{2\epsilon_{2}}{\dot{g}}^{2}+\frac{\epsilon_{2}}{2}{\tilde{g}}^{2}$$

Consequently, it gives

(30) $${\dot{V}}_{2}\leq -kx_{e}^{2}-\left(c_{min}-\frac{1}{2\epsilon_{1}}\right)y_{e}^{2}-\left(k_{g}-\frac{\epsilon_{1}}{2}-\frac{\epsilon_{2}}{2}\right){\tilde{g}}^{2}+\frac{1}{2\epsilon_{2}}{\dot{g}}^{2}\;\leq -hV_{2}+\frac{1}{2\epsilon_{2}}g_{*}^{2}$$

where $h=min\left\{k,c_{min}-\frac{1}{2\epsilon_{1}},k_{g}-\frac{\epsilon_{1}}{2}-\frac{\epsilon_{2}}{2}\right\}>0$. □

Therefore, $V_{2}\leq e^{-ht}V_{2}\left(0\right)+\frac{1}{2h\epsilon_{2}}g_{*}^{2}$. Obviously, $V_{2}$ is bounded and has exponential decay, and the improved ELOS guidance system is uniformly ultimately bounded [39].

## 4. Results

**Figure 3:**
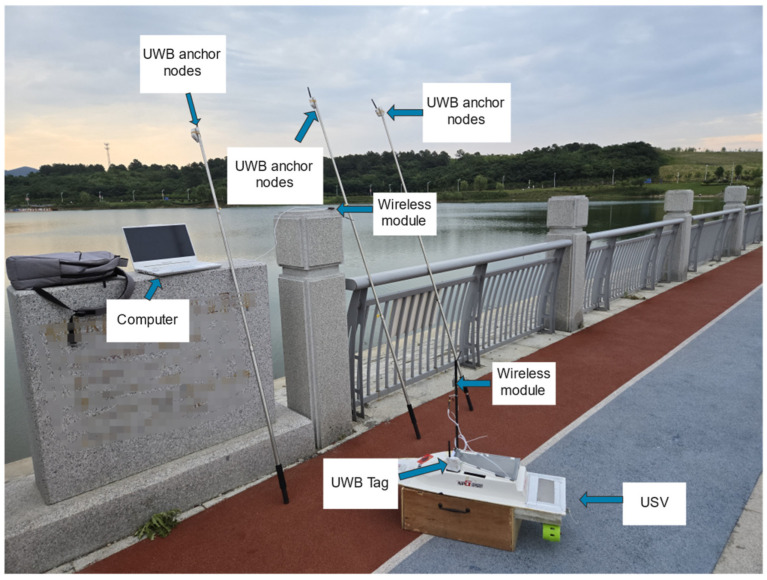
The experimental system for a USV.

In this section, Figure 3 shows the sensor applications related to the self-developed USV. Then, several guidance laws are simulated, and the simulation results are analyzed. Finally, the experimental results of the USV verify the effectiveness of the ELOS guidance law.

To better illustrate the effectiveness of the improved ELOS guidance law, a comparative study of ELOS, ILOS, and ALOS guidance laws was performed. 

The ELOS guidance law proposed in [23] is as follows:

(31) $$\left\{\begin{array}{c} \dot{p}=-kp-k^{2}y_{e}-k\left[U\sin\left(\psi_{d}-\alpha_{k}\right)-{\dot{\alpha }}_{k}x_{e}\right]\\ \hat{g}=p+ky_{e} \end{array}\right.$$

(32) $$\hat{\beta }=\frac{\hat{g}}{U\cos\left(\psi_{d}-\alpha_{k}\right)}$$

(33) $$\psi_{d}=\alpha_{k}\left(s\right)+\arctan\left(-\frac{y_{e}}{\Delta }-\hat{\beta }\right)$$

(34) $$\dot{s}=\frac{U\cos\left(\psi -\alpha_{k}\right)+\kappa x_{e}}{\sqrt{{{x^{\prime }}_{k}}^{2}\left(s\right)+{{y^{\prime }}_{k}}^{2}\left(s\right)}}$$

The ILOS guidance law proposed in [27] is as follows:

(35) $$\left\{\begin{array}{c} \psi_{d}=\alpha_{k}(s)-\arctan(\frac{y_{e}}{\Delta }+k_{int}y_{\mathrm{int}})\\ {\dot{y}}_{\mathrm{int}}=\frac{Uy_{e}}{\sqrt{\Delta^{2}+(y_{e}+\mu y_{\mathrm{int}}{)}^{2}}} \end{array}\right.$$

The ALOS guidance law proposed in [27] is as follows:

(36) $$\left\{\begin{array}{c} \psi_{d}=\alpha_{k}\left(s\right)+\arctan\left(-\frac{y_{e}}{\Delta }-\hat{\beta }\right)\\ \dot{\hat{\beta }}=\frac{k_{a}U\Delta y_{e}}{\sqrt{\Delta^{2}+{\left(y_{e}+\Delta \hat{\beta }\right)}^{2}}} \end{array}\right.$$

The improved ELOS guidance law (14) is applied to the simulation by selecting the control parameters as follows: Δ = 8 m, *k* = 10, *κ* = 12. A PID controller is used to control the course of the USV.

(37) $$\tau_{r}=-k_{p}\psi_{e}-k_{i}\int_{0}^{t}\psi_{e}-k_{d}{\dot{\psi }}_{e}$$

where $\psi_{e}=\psi -\psi_{d}$, $k_{p}$, $k_{i}$ and $k_{d}$ is the PID controller is used to control the course of USV.

### 4.1. USV and Shipborne Sensors

**Figure 4:**
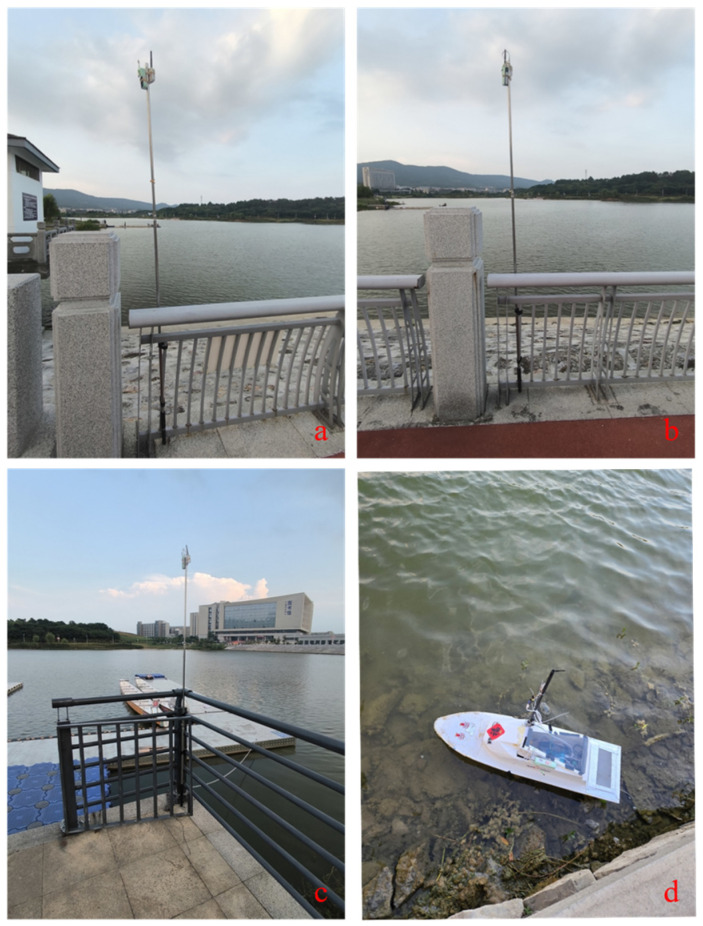
The experiment settings of the experimental scene, where (**a**–**d**) are, respectively, base station 0, base station 1, base station 2, and shipborne label 0.

Figure 3 illustrates the combination of the UWB and USV. The UWB base station and the UWB tag cooperate to form the UWB positioning system. The three UWB base stations were fixed at specific locations on the shore, and the UWB tags were fixed to the side of the USV. Furthermore, the experiment settings of the experimental scene are shown in Figure 4. The adopted UWB positioning algorithm was built on [43]. For the convenience of shooting, the three base stations were put together with the USV, while the coordinates of the three base stations were (0, 0), (33.5, 0), and (−10.2, 83.4), respectively. In addition, the attitude angle and acceleration information of the USV was determined using an IMU device. Moreover, after obtaining the USV’s attitude angle, acceleration, and position information, the surge velocity *u* and sway velocity *v* were obtained using Kalman filtering [44].

The accuracy of the positioning system combined with radar and satellite images was about 9.77 m [45]. In contrast, the accuracy of the general GPS positioning system was a little better, about 2 m [23]. However, the accuracy of UWB positioning system could reach 10 cm. This shows that the accuracy of USV positioning was significantly improved after the introduction of UWB technology.

### 4.2. Simulation Results

In this section, MATLAB R2021a simulation results are presented to verify the performance of the proposed guidance law for the USV path-following system. It is worth noting that in the USV experiment, the control system of USV was a discontinuous, discrete, nonlinear system due to the existence of sensor sampling time. Hence, the simulation experiment was designed as a discrete, nonlinear system with a sampling time of 0.1 s to better fit the real situation. Furthermore, the guidance laws (14), (33), (35), (36) and control law (37) are accordingly changed into corresponding discrete forms.

For simulation the dynamic model of USV was used as follows:

(38) $$\left\{\begin{array}{l} \dot{u}=f_{u}+\frac{\tau_{u}}{m_{u}}+\frac{\tau_{d1}}{m_{11}}\\ \dot{v}=f_{v}+\frac{\tau_{d2}}{m_{v}}\\ \dot{r}=f_{r}+\frac{\tau_{r}}{m_{r}}+\frac{\tau_{d3}}{m_{r}} \end{array}\right.$$

where

(39) $$\left\{\begin{array}{c} f_{u}=\frac{m_{\nu }}{m_{u}}vr-\frac{d_{u1}}{m_{u}}u,\\ f_{\nu }=\frac{m_{u}}{m_{\nu }}ur-\frac{d_{\nu 1}}{m_{\nu }}\nu ,\\ f_{r}=\frac{m_{u}-m_{v}}{m_{r}}uv-\frac{d_{r1}}{m_{r}}r \end{array}\right.$$

The relevant parameters are detailed in Table 1.

The improved ELOS, ELOS, ALOS, and ILOS guidance laws were simulated to highlight the effectiveness and superiority of the proposed guidance law in the presence of disturbances. The simulations were conducted at a speed of 1 m/s, with PID controllers used to control the heading of the USV. Parameters were set to compare the performance of these four guidance laws and to evaluate their effectiveness. 

The reference path was defined by parametric equations similar to those in [46].

(40) $$\left\{\begin{array}{c} x=10\sin(0.1s)+s\\ y=s \end{array}\right.$$

An oscillation system based on the second order that gets hold of Gaussian noise was established to simulate wind and waves, as shown in (41).

(41) $$h\left(\omega \right)=\frac{0.4198\omega }{\omega^{2}+0.3638\omega +0.3675}$$

Table 2 demonstrates the parameters of the controllers. To ensure a fair comparison, the controller parameters were manually tuned to an optimal state.

The following performance criteria were used to compare the performance of these guidance laws.

(42) $$\left\{\begin{array}{l} MAE=\frac{1}{t_{\infty }-t_{0}}\int_{t_{0}}^{t_{\infty }}\left|x_{k}-x\left(t\right)\right|dt\\ MIA=\frac{1}{t_{\infty }-t_{0}}\int_{t_{0}}^{t_{\infty }}\left|u\left(t\right)\right|dt\\ MTV=\frac{1}{t_{\infty }-t_{0}}\int_{t_{0}}^{t_{\infty }}\left|u\left(t\right)-u\left(t-1\right)\right|dt \end{array}\right.$$

The mean absolute error (MAE) reflects the average cross-track error. A smaller MAE indicates a better tracking effect of the guidance law. The mean absolute integral (MIA) reflects energy consumption. A smaller MIA indicates that the USV consumes less energy under this guidance law. The mean total variation (MTV) reflects the smoothness of the guidance law. The smaller the MTV, the better the smoothness of the controller under the action of this guidance law [47]. As exhibited in Table 3, the results of the performance analysis metrics are shown in Table 3.

Performance analysis in Table 3 shows that the performance of the improved ELOS was the best, followed by ELOS, then ALOS, and ILOS was the worst. This is also consistent with the simulation results.

**Figure 5:**
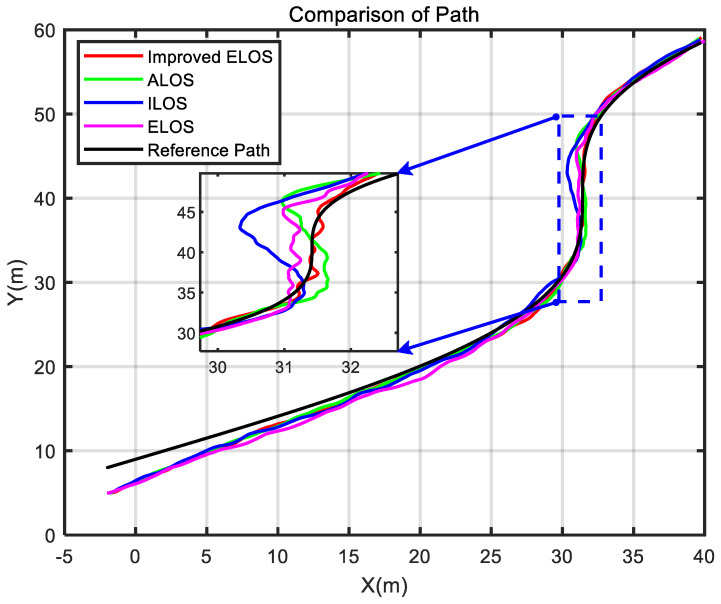
Comparison of USV path tracking under different guidance laws.

**Figure 6:**
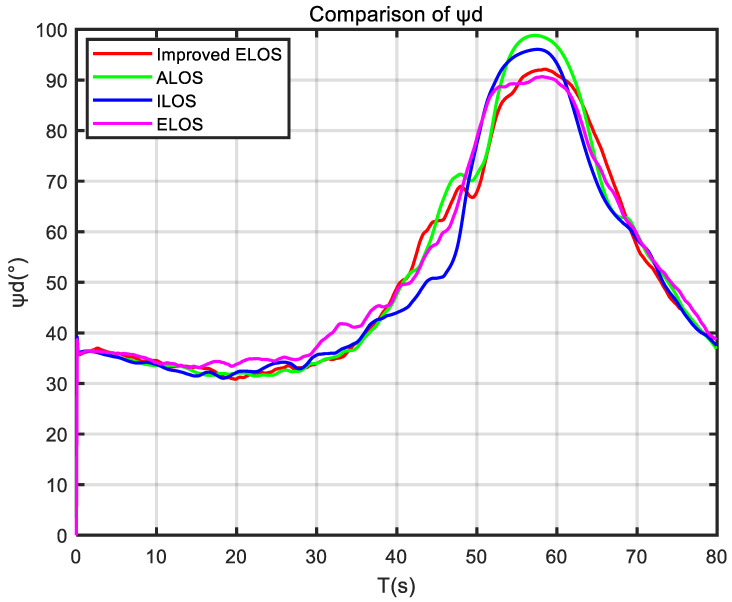
Comparison of $\psi_{d}$ calculated by different guidance laws.

**Figure 7:**
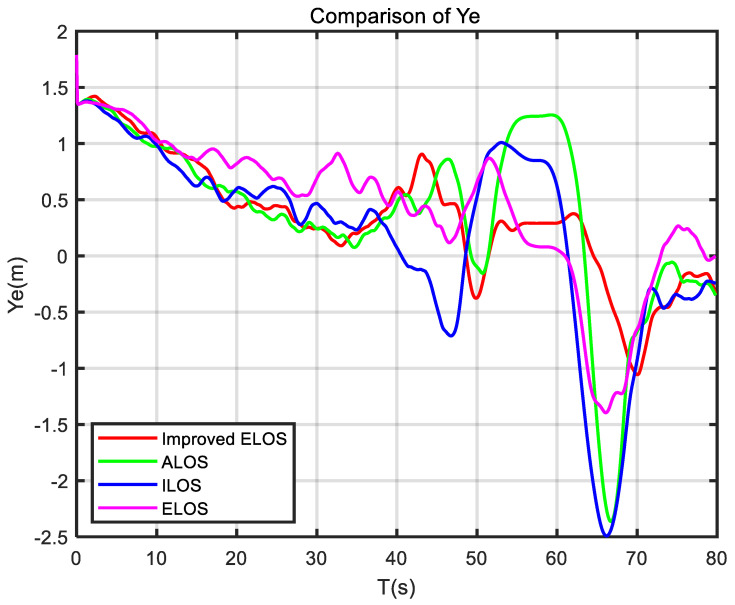
Comparison of cross-track error Ye under different guidance laws.

The simulation results are plotted in Figure 5, Figure 6 and Figure 7. As demonstrated in Figure 5, when the USV navigated in a straight line, all four algorithms could track the path closely with little difference between them. However, at the turning points, the improved ELOS algorithm performed well in tracking the path. In contrast, the ELOS, ALOS, and ILOS algorithms demonstrated different error levels. While ELOS was similar to the improved ELOS, ALOS and ILOS showed larger errors, indicating significant sideslip angles present in these two guidance laws. Figure 7 demonstrates that the cross-tracking error of improved ELOS compared to ELOS was smaller, attributed to the effective real-time estimation and compensation of sideslip angle beta in both improved ELOS and ELOS. Due to the integration component in ILOS being prone to integral saturation, its cross-tracking error was the highest among the four algorithms. On the other hand, the ALOS guidance law, which uses an adaptive method to estimate the sideslip angle, does not require an integration term, thus avoiding integral saturation and resulting in smaller cross errors compared to ILOS. The comparison of the average cross-tracking errors among the four guidance laws also reflected this observation.

Figure 6 demonstrates that improved ELOS, ELOS, and ALOS can quickly respond to changes in the curve, adjusting the desired heading angle promptly. However, due to the time required for integration in ILOS, it could not adjust the desired heading angle on time.

### 4.3. Experimental Results

To further verify the effectiveness and practicability of the improved ELOS guidance law, a straight path-following experiment was carried out to show the tracking performance of the improved ELOS guidance law. An underactuated USV controlled by a Raspberry PI was used as an experimental platform for the guidance law. The experimental site was a reservoir within the school. Moreover, on the day of the experiment, the wind speed was about 1.6 m/s and the wave height was about 5 cm. 

**Figure 8:**
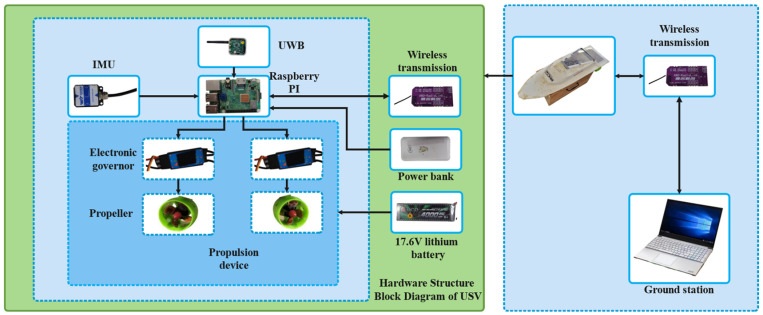
The USV control system structure.

The USV control system structure is demonstrated in Figure 8.

The main control board for this project was the Raspberry Pi 3B, a single-board computer based on ARM architecture. It comes with a 1.2 GHz quad–core ARM Cortex–A53 processor, 1 GB of RAM, and integrated 802.11n wireless networking, and Bluetooth 4.1. The Raspberry Pi 3B supports various connectivity options.

The surge velocity *u* and sway velocity *v* of the USV were not directly available. Therefore, IMU was used to obtain the attitude angle and acceleration information of USV and was combined with the positioning information of UWB system to conduct Kalman filtering to obtain the surge velocity *u* and sway velocity *v*.

The communication module employed efficient algorithms for bidirectional data transmission without requiring manual switching or additional operations, ensuring minimal data latency in milliseconds and support for long-distance, strong signal transmission at a default baud rate of 500,000.

The thrusters were equipped with fixed twin-propellers and two DC motors with a power range of 30–200 W, operating at a voltage of 12–24 V and a current of 13 A. The motor speed was controlled through PWM waves generated by the Raspberry Pi. Experimental tests determined the PWM range of 40–110 μs for proper motor operation, with a maximum of 110 μs for forward rotation, and 40 μs for reverse rotation. When the PWM signal was set to 76 μs [48], the relationship between PWM and thrust control is described as (43).

(43) $$T_{pwm}=\frac{T_{N}}{T_{max}}\times 30+76$$

where $T_{pwm}$ represents the calculated PWM, $T_{N}$ is the required thrust, and $T_{max}$ is the maximum thrust generated by the thruster. Furthermore, the relationship equations between forces $\tau_{u}$, moment $\tau_{r}$ and the thrusts were obtained as below:

(44) $$\left\{\begin{array}{c} \tau_{u}=T_{R}+T_{L}\\ \tau_{r}=\frac{\left(T_{R}-T_{L}\right)B}{2} \end{array}\right.$$

where $T_{L}$, $T_{R}$ represent the thrust of the left and right motors, respectively. And B is the distance of the motor to the center shaft of the ship. Table 4 demonstrates the parameters of the USV.

The attitude angle and acceleration information of the USV was determined using an IMU device. Moreover, a UWB positioning system was applied to determine the position of the USV. Moreover, after obtaining the USV’s attitude angle, acceleration, and position information, the surge velocity *u* and sway velocity *v* were obtained using Kalman filtering.

Table 5 displays the parameters of the improved ELOS algorithm and PID controller utilized in the experiments.

**Figure 9:**
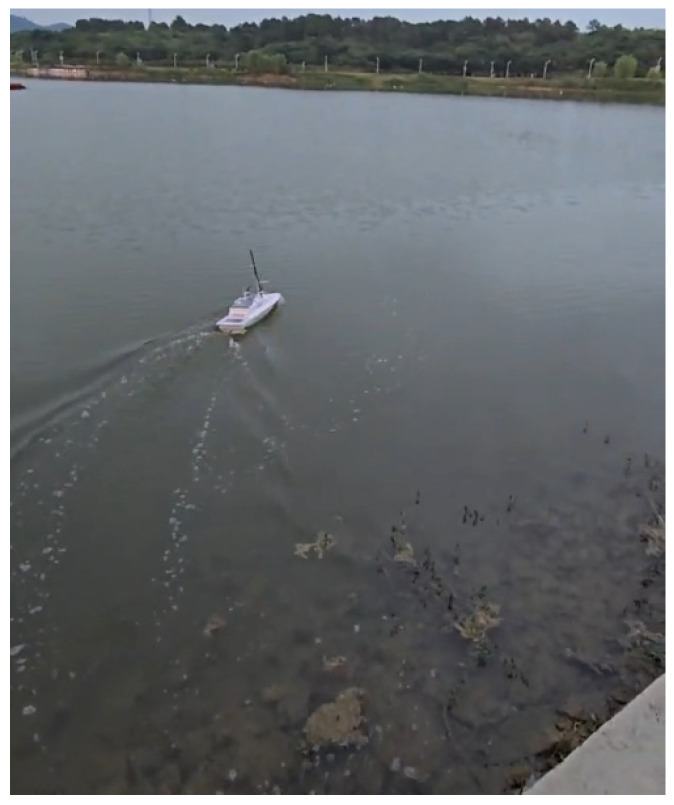
Real-world test.

**Figure 10:**
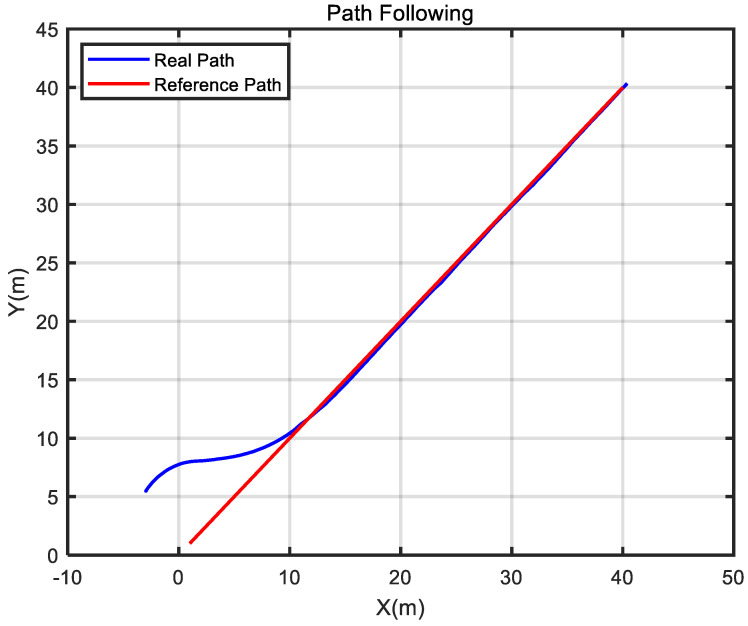
Comparison of the USV actual path with the reference path.

**Figure 11:**
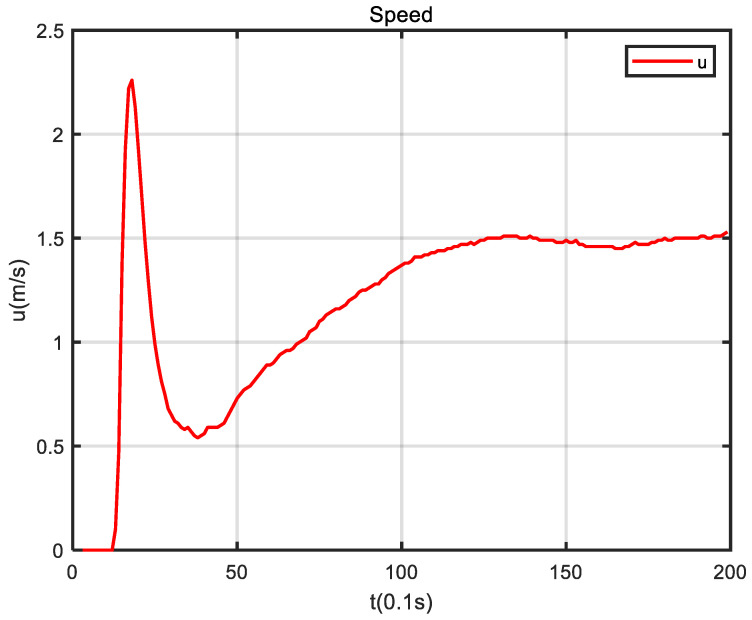
The USV surge velocity changes.

**Figure 12:**
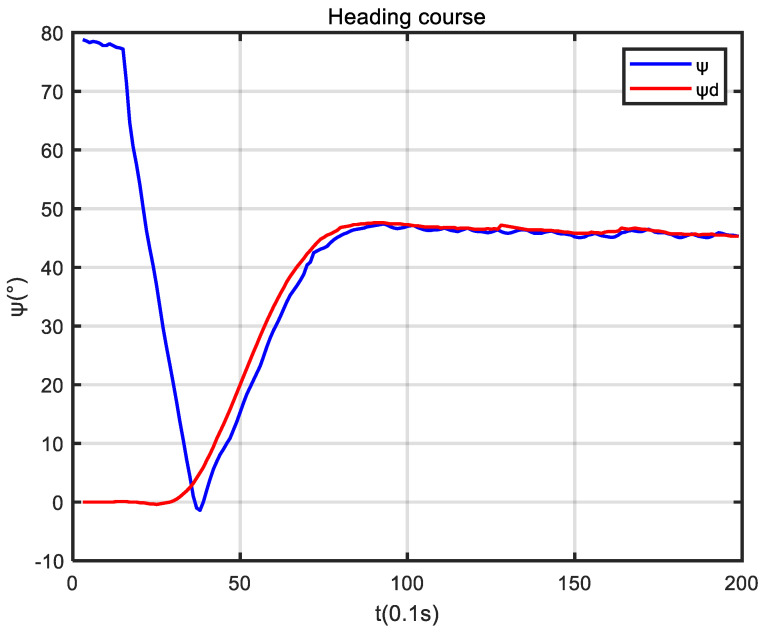
The USV heading course changes.

In the experiment, the initial heading angle of the USV was set to 90°, and the initial position was (−3, 5.5). Figure 9 shows that USV conducted path tracking from the starting position in the experiment. As seen in Figure 10, based on the reference heading angle calculated by the improved ELOS guidance law, and under the control of the PID heading controller, the USV started from the starting position and gradually approached the set reference path after turning. After some time, the USV reached the reference path and travelled along it. When navigating along the reference path, the error between the USV and the reference path was small, which verifies the validity of the guidance law proposed in this paper. Figure 11 shows that the speed obtained from the Kalman filter initially increased, then decreased, and subsequently increased and stabilized to 1.5 m/s. It can be seen from Figure 12 that under PID control, the USV’s heading angle changed rapidly from the initial angle to the expected angle calculated by the guidance law and could keep up with the change of the expected angle.

## 5. Conclusions

The improved ELOS guidance law maintains the clarity and comprehensibility of the classical ELOS control law, while accurately identifying the sideslip angle of the USV. Based on the error dynamic system of the along–track error and cross–track error, the reduced order ESO is used to estimate the time-varying sideslip angle without small angle approximation, which ensures robustness with the uncertainty of track errors and observation errors. The proposed method effectively alleviates the undesired drift error caused by external disturbances, thus improving the path-following performance. In the simulations, the average cross–track error of the improved ELOS guidance law is 0.5384 m, respectively 19.6% and 24.9% lower than that of ELOS and ILOS. In the experiment, the USV sailed along the reference path under the improved ELOS guidance law, which verifies the effectiveness of the proposed guidance law. In summary, the improved ELOS has obvious performance improvement, compared with guidance laws such as ELOS and ILOS.

## Figures and Tables

**Table 1:** The relevant parameters.

Parameters	Value
$m_{u}\;(kg)$	22.8
$m_{\nu }\;(kg)$	50.9
$m_{r}\;(kg)$	11.6
$d_{u1}\;(Nm/s)$	29.4
$d_{v1}\;(Nm/s)$	0
$d_{r1}\;(Nm/s)$	1.6

**Table 2:** Parameters of the individual guidance laws.

Methods	Parameters	Value
Improved ELOS	Δ	8
	$k_{g}$	10
	κ	12
ELOS	Δ	8
	k	10
	κ	12
ALOS	Δ	8
	$k_{a}$	0.1
ILOS	Δ	8
	$k_{int}$	0.01
	μ	1

**Table 3:** Comparison of performance analysis specifications.

Guidance Laws	MAE	MIA	MTV
Improved ELOS	0.5384	1.7779	0.4383
ELOS	0.6696	1.9118	0.4750
ALOS	0.6891	1.8340	0.4688
ILOS	0.7168	1.8760	0.4538

**Table 4:** Parameters of the USV.

Parameters	Value
Length overall (m)	1.02
Length on the water (m)	0.91
Beam overall (m)	0.18
Mass (kg)	5.5

**Table 5:** The parameters of the algorithm.

Algorithm	Parameters	Value
PID	$K_{p}$	5
	$K_{i}$	0.01
	$K_{d}$	0.01
Improved ELOS	Δ	6
	$k_{g}$	10
	κ	12

## Data Availability

Data will be made available on request.
